# Self-compassion and Psychological Distress in Adolescents—a Meta-analysis

**DOI:** 10.1007/s12671-017-0850-7

**Published:** 2017-11-25

**Authors:** Imogen C. Marsh, Stella W. Y. Chan, Angus MacBeth

**Affiliations:** Department of Clinical and Health Psychology, School of Health in Social Science, University of Edinburgh, Rm 3.06A, Doorway 6, Medical Quad, Teviot Place, Edinburgh, EH8 9AG UK

**Keywords:** Self-compassion, Adolescence, Anxiety, Depression, Stress, Meta-analysis

## Abstract

Research indicates that self-compassion is relevant to adolescents’ psychological well-being, and may inform the development of mental health and well-being interventions for youth. This meta-analysis synthesises the existing literature to estimate the magnitude of effect for the association between self-compassion and psychological distress in adolescents. Our search identified 19 relevant studies of adolescents (10–19 years; *N* = 7049) for inclusion. A large effect size was found for an inverse relationship between self-compassion and psychological distress indexed by anxiety, depression, and stress (*r* = − 0.55; 95% CI  − 0.61 to − 0.47). The identified studies were highly heterogeneous, however sensitivity analyses indicated that correction for publication bias did not significantly alter the pattern of results. These findings replicate those in adult samples, suggesting that lack of self-compassion may play a significant role in causing and/or maintaining emotional difficulties in adolescents. We conclude that self-compassion may be an important factor to target in psychological distress and well-being interventions for youth.

## Introduction

Adolescence is a time of rapid biological, cognitive, and social change. These normative developmental changes may contribute to some mental health issues, such as elevated stress levels (Byrne et al. 2007). The global prevalence of mental health problems in youth is estimated as 10–20% (Patel et al. 2007); whilst mental health problems in youth predict poor educational achievement, physical ill health, substance misuse, and conduct problems in later life (Patel et al. 2007). It is estimated that 15–30% of disability-adjusted life years are lost to early mental health problems (Kieling et al. 2011), and thus present a significant burden to the global economy (Patel et al. 2007). The most common mental health issues experienced in adolescence are stress, anxiety, and depression (Cummings et al. 2014).

Stress in adolescence is significantly related to anxiety, depression, and suicide (Byrne et al. 2007; Grant 2013). Females are more vulnerable to stress (Parker and Brotchie 2010), which may be related to the increased prevalence of anxiety and depression observed in this group. Similarly, it has been reported that older adolescents are more vulnerable to stress, although it has been suggested that this is more related to the increasing demands on the individual and improving intellectual capacity to consider an uncertain future, than to age itself (Byrne et al. 2007).

Anxiety disorders are common in youth, with a lifetime prevalence of 15–20% (Beesdo et al. 2009). They hinder psychosocial development and are associated with serious comorbid difficulties including depression and suicidality (Cummings et al. 2014). Female adolescents have been reported to be two to three times more likely to experience anxiety (Beesdo et al. 2009).

The lifetime prevalence of depression in 13- to 18-year-olds has been reported as 11% (Hankin 2015). It is the third most significant factor related to suicide completion in the adolescent population, and is highly predictive of further psychological difficulties in adulthood, such as anxiety disorders, substance misuse, and bipolar disorder (Thapar et al. 2012). Furthermore, it has been hypothesised that the substantial increase in depression during adolescence is related to a range of physical and psychosocial changes (Spear 2000). As with findings regarding anxiety, female adolescents are twice as likely to experience depression as their male peers (Thapar et al. 2012).

The global impact of youth psychological distress highlights the need to identify mechanisms of change to inform effective psychological health promotion and interventions for this population. One potential mechanism of change that has been indicated in adult and adolescent samples is self-compassion (MacBeth and Gumley 2012; Xavier et al. 2016b). The concept of self-compassion is rooted in Buddhist philosophy (where self-compassion is considered to be identical to compassion towards others, merely turned inward to the self (Neff 2004). Neff’s (2003a) dimensional model of self-compassion proposes that self-compassion exists on a spectrum from high to low (Neff 2016), and that self-compassion comprises three spectra (each with opposing poles): self-kindness vs. critical self-judgement, common humanity vs. isolation, and mindfulness vs. over-identification. The construct of self-kindness encapsulates an individual’s ability to respond to their own suffering with warmth and the desire to alleviate their own pain (Neff and Dahm 2013). Common humanity reflects an individual’s capacity to recognise that all humans share similar internal experience and that their suffering is not unique. Mindfulness consists of the ability to dispassionately consider aversive experience and maintain distance between the self and emotions (Neff and Dahm 2013). The self-compassion scale (SCS; Neff 2003b) is the most prevalent standardized measure of self-compassion, and thus the majority of the literature examining self-compassion draws explicitly on Neff’s dimensional model.

Another model of (self) compassion has been developed by Gilbert (2009). Gilbert’s (2009) model frames compassion as the result of adaptive capacities shaped by evolution, and emphasizes the physiological and neurological correlates of mental and emotional states. The Gilbert model is constructed on the premise that the “compassion system” is separate to the “critical system” (Gilbert 2014), suggesting that these constructs should be measured independently, in contrast to Neff’s (2003b) dimensional conceptualization. However, both Neff and Gilbert’s models propose that self-compassion is a relational state characterized by kindness and empathy, and are complementary frameworks for understanding the concept of compassion towards the self and others (MacBeth and Gumley 2012).

In adult samples, self-compassion has been shown to account for a significant degree of variance in psychological well-being, and is predictive of lower symptom severity in anxiety and depression, as well as higher quality of life (Neff et al. 2007; Van Dam et al. 2011). Self-compassion and psychopathology have also been shown to be significantly negatively correlated, with a large effect size, in adult clinical and non-clinical populations (MacBeth and Gumley 2012; Zessin and Garbade 2015). Whilst the research base regarding self-compassion in the adolescent population is still emerging (Xavier et al. 2016b), findings to date appear to mirror those in adult samples. Several studies have shown that female adolescents have lower levels of self-compassion than their male counterparts (Bluth and Blanton 2014; Castilho et al. 2017; Sun et al. 2016). Age has also been shown to interact with gender, with older female adolescents (above 14 years) reporting lower levels of self-compassion than younger females and male adolescents (Bluth and Blanton 2015; Bluth et al. 2017; Muris et al. 2016). There are also indications that self-compassion may have a different ‘action’ in males and females: Bluth and Blanton (2015) reported that in males self-compassion only mediated the relationship between mindfulness and negative affect, whereas self-compassion also mediated the relationship between mindfulness and perceived stress in their female counterparts.

As in adult samples, self-compassion has been identified as a predictor of well-being in adolescents. Low self-compassion has been shown to be predictive of elevated depressive symptoms (Trollope 2009; Williams 2013), elevated psychological distress, problem alcohol use, and serious suicide attempts (Tanaka et al. 2011). In a naturalistic longitudinal study, Zeller et al. (2015) found that a higher level of self-compassion at baseline was predictive of lower levels of psychopathology (depression, post-traumatic stress, panic, and suicidality) following a traumatic event in a sample of adolescents.

Additionally, self-compassion has been identified as a “buffer” against a range of negative psychological and physical health outcomes in adolescent populations. Játiva and Cerezo (2014) found that self-compassion acts as a buffer between negative life experiences (such as victimisation) and poor psychological outcomes in disadvantaged youths. Trollope (2009) and Williams (2013) both identified a significant inverse relationship between self-compassion and depression, reporting preliminary indications that self-compassion mediates the relationship between stressful life-events and depressive symptoms (Trollope 2009) and social rank and depression (Williams 2013). In relation to this, Marshall et al. (2015) found that high self-compassion buffered the detrimental effect of low self-esteem on mental health in this population. Castilho et al. (Castilho et al. 2017) report findings which suggest that self-compassion and emotional intelligence are key regulatory process in protecting against depressive symptoms in adolescents. It also appears that self-compassion could reduce risky behaviour fuelled by psychological distress in this population. The relationship between depressive symptoms and non-suicidal self-injury (NSSI; Xavier et al. 2016b) and peer victimisation and NSSI (Jiang et al. 2016) has been shown to be buffered by self-compassion, as has the relationship between chronic academic stress and negative affect (Zhang et al. 2016).

High self-compassion has even been shown to ameliorate markers of physiological stress in response to the Trier Social Stress Test (Bluth et al. 2016b). Similarly, in a sample of adolescents with chronic headache—where depression was found to be the most significant risk factor for headache-related disability—self-compassion was identified as a potential moderator of the depression-headache disability relationship (Kemper et al. 2015).

The research evidence thus far indicates the potential validity of self-compassion as a point of intervention in psychological well-being for the adolescent population, as in adult samples (Barnard and Curry 2011). Indeed, in adolescent samples, interventions which explicitly teach self-compassion skills have been found to successfully elevate levels of self-compassion (Galla 2016; Bluth et al. 2016a). Participation in these programmes and elevation of self-compassion was associated with reduced rumination (Galla 2016), reduced depressive symptoms, and increased positive affect and life satisfaction (Bluth et al. 2016a; Galla 2016). Self-compassion may be relevant to adolescents’ psychological well-being, as it is in adult populations (Marshall et al. 2015). As yet, research regarding self-compassion and psychological well-being in adolescents has not been synthesised using systematic review or meta-analytic approaches, therefore the potential value of self-compassion to this population is not yet truly understood or quantified. Consequently, the objectives of this meta-analysis were threefold. First, we sought to estimate the magnitude of association between self-compassion and psychological distress in adolescent populations. We hypothesised that self-compassion and psychological distress would be negatively correlated in adolescents, in line with previous findings in adults (MacBeth and Gumley 2012). Second, we investigated potential sources for the heterogeneity within effect size estimates. Third, we aimed to systematically assess the quality of research on self-compassion in adolescent mental health.

## Method

### Literature Search

**Fig. 1:**
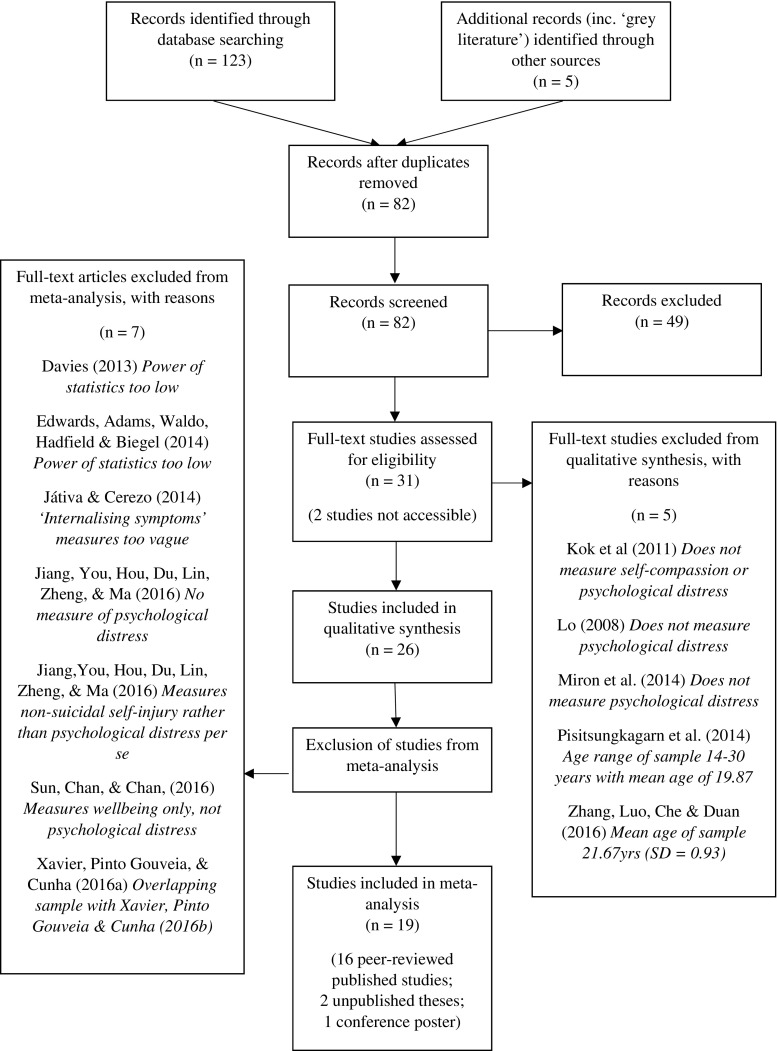
Systematic search and selection process (PRISMA; Moher et al. 2009)

A systematic review was performed using PRISMA criteria (Moher et al. 2009). Literature searches were conducted in four bibliographic databases: EMBASE, MEDLINE, PsychINFO, and ProQuest Dissertations and Theses Global. Google Scholar was employed to search for peer-reviewed, in-press research that were available online but not via databases, and other “grey literature” such as unpublished/unregistered theses and conference abstracts/scientific posters. The following search terms were used in a two-component strategy: component 1 (self-compassion) and component 2 (adolescent or young adult or child). Figure 1 depicts the search and selection process.

### Inclusion and Exclusion Criteria

Studies were considered eligible for inclusion if the participants were aged between 10 and 19 years, and the study included valid and reliable measures of both self-compassion and psychological distress (e.g., depression, anxiety, and stress). These age parameters were chosen to provide a sample which reflects the World Health Organisation’s definition of “adolescence” (Sacks 2003), taking into account the chronological ages usually associated with developmental (pubertal and social) changes of adolescence. Studies were excluded if they were not available in English. To ensure reliability of the review process, four articles (21%) in the final data set were assessed by an independent reviewer. A 100% agreement on inclusion was reached between the first author (ICM) and the independent reviewer.

### Sample of Studies

Implementation of the search strategy and inclusion/exclusion criteria identified a final set of 19 studies, representing 19 cohorts (*N* = 7074) for the meta-analytic sample (see Table 1). The systematic search was conducted in December 2016. All studies identified were published between 2009 and the end of 2016; 16 were peer-reviewed published articles, 2 were unpublished theses, and 1 study was reported in the form of a conference poster. The included studies reported 7 effect sizes for the anxiety/self-compassion relationship, 13 effect sizes for the depression/self-compassion relationship, and 11 effect sizes for the stress/self-compassion relationship. Table 2 provides a summary of study characteristics by aspect of psychological distress reported.

**Table 1:** Studies included in meta-analysis (*n* = 19)

Authors and year	Title	*N*	Anxiety	Depression	Stress
Barry et al. (2015)	Adolescent self-compassion: associations with narcissism, self-esteem, aggression, and internalising symptoms in at-risk males.	251	✓	✓	✗
Bluth and Blanton (2014)	Mindfulness and self-compassion: exploring pathways to adolescent emotional well-being.	67	✗	✗	✓
Bluth and Blanton (2015)	The influence of self-compassion on emotional well-being among early and older adolescent males and females.	90	✗	✗	✓
Bluth et al. (2017)	Age and gender differences in the associations of self-compassion and emotional well-being in a large adolescent sample.	765	✓	✓	✓
Bluth et al. (2016a)	Making friends with yourself: a mixed methods pilot study of a mindful self-compassion program for adolescents.	34	✓	✗	✓
Bluth et al. (2015)	A pilot study of a mindfulness intervention for adolescents and the potential role of self-compassion in reducing stress.	28	✗	✗	✓
Bluth, Roberson, Gaylord, Faurot, Grewen, Arzon & Girdler (2016)	Does self-compassion protect adolescents from stress?	28	✓	✗	✓
Castilho et al. (2017)	Self-compassion and emotional intelligence in adolescence: a multigroup mediational study of the impact of shame memories on depressive symptoms.	1101	✗	✓	✗
Cunha et al. (2013)	Early memories of positive emotions and its relationships to attachment styles, self-compassion and psychopathology in adolescence.	651	✓	✓	✓
Galla (2016)	Within-person changes in mindfulness and self-compassion predict enhanced emotional well-being in healthy, but stressed adolescents.	132	✗	✓	✓
Kemper et al. (2015)	What factors contribute to headache-related disability in teens?	29	✓	✓	✓
Marshall et al., 2015	Self-compassion protects against the negative effects of low self-esteem: a longitudinal study in a large adolescent sample.	2448	✗	✗	✓
Neff and McGehee (2010)	Self-compassion and psychological resilience among adolescents and young adults.	235	✓	✓	✗
Stolow et al. (2016)	A prospective examination of self-compassion as a predictor of depressive symptoms in children and adolescents.	223*	✗	✓	✗
Tanaka et al. (2011)	The linkages among childhood maltreatment, adolescent mental health, and self-compassion in child welfare adolescents.	117	✗	✓	✓
Trollope (2009)	Stressful life-events and adolescent depression: the possible roles of self-criticism and self-compassion	107	✗	✓	✗
Williams (2013)	Examining the moderating effects of adolescent self-compassion on the relationship between social rank and depression.	119	✗	✓	✗
Xavier et al. (2016b)	The protective role of self-compassion on risk factors for non-suicidal self-injury in adolescence.	643	✓	✗	✗
Zeller et al. (2015)	Self-compassion in recovery following potentially traumatic stress: longitudinal study of at-risk youth.	64	✗	✓	✗
Totals		7132	8	12	11

*The study authors provided data regarding a subset of participants in their study, in order to comply with the age parameters of this meta-analysis

**Table 2:** Summary of study effect sizes included in meta-analysis (*n* = 19) by psychological distress outcome type

Study	Sample *N*	Symptom measure	Participants	Study design	Age: mean; S.D.; range	Gender ratio (F/M)	*r*
Anxiety							
Barry et al. (2015)	251	SCS; PIY	Adolescents in residential programme	Cross-sectional	16.78; 0.73; 16–18.	0/251	− 0.32
Bluth et al. (2017)	765	SCS-SF; STAI-T; SMFQ; PSS	Secondary school Pupils	Cross-sectional	14.6; unknown; 11–19	405/360	− 0.53
Bluth et al. (2016a)	34	CAMM; PANAS; SCS-SF; SMFQ; PSS; STAI	Adolescent volunteers	Experimental	14.64; unknown; 14–17	26/8	− 0.39
Bluth, Roberson, Gaylord, Faurot, Grewen, Arzon & Girdler (2016)	28	PSS; SCS; SSAI	Adolescent volunteers	Experimental	14.93; 1.63; 13–18	22/6	− 0.47
Cunha et al. (2013)	651	SCS; DASS-21	Secondary school pupils	Cross-sectional	15.89; 1.99; 12–19.	321/330	− 0.33
Kemper et al. (2015)	29	PSS; CAMS-R	Adolescents with chronic headache	Cross-sectional	14.8; 2.0; unknown	20/9	− 0.42
Neff and McGehee (2010)	235	SCS; STAI-T	Secondary school pupils	Cross-sectional	15.2; unknown; 14–17.	113/122	− 0.73
Depression							
Barry et al. (2015)	251	SCS; PIY	Adolescents in residential programme	Cross-sectional	16.78; 0.73; 16–18.	0/251	− 0.27
Bluth et al. (2017)	765	SCS-SF; STAI-T; SMFQ; PSS	Secondary school pupils	Cross-sectional	14.6; unknown; 11–19	405/360	− 0.51
Castilho et al. (2017)	1101	SCS-A; CDI	Secondary school pupils	Cross-sectional	15.94; 1.21; unknown	632/469	*f* − 0.62
							*m* − 0.52
Cunha et al. (2013)	651	SCS; DASS	Secondary school pupils	Cross-sectional	15.89; 1.99; 12–19.	321/330	− 0.46
Galla (2016)	132	SCS-SF; PSS; CES-D	Healthy “stressed” adolescent volunteers	Longitudinal	16.76; 1.48; unknown	80/52	− 0.56
Kemper et al. (2015)	29	PSS; CAMS-R	Adolescents with chronic headache	Cross-sectional	14.8; 2.0; unknown	20/9	− 0.67
Neff and McGehee (2010)	235	SCS; BDI	Secondary school pupils	Cross-sectional	15.2; unknown; 14–17.	113/122	−0.60
Stolow et al. (2016)	223	CDI; SCS	Secondary school pupils	Longitudinal	14.2; unknown; 12–16	124/99	− 0.59
Tanaka et al. (2011)	117	SCS; CES-D; GHQ-12	Adolescents in CPS	Cross-sectional	18.1; unknown; 16–20.	64/53	−0.37
Trollope (2009)	107	SCS; IHSSRLE	Secondary school pupils	Cross-sectional	12.74; unknown; 12–14.	54/53	− 0.64
Williams (2013)	119	SCS; CDI	Secondary school pupils	Cross-sectional	16.3; unknown; 15.1–18.7.	72/47	− 0.60
Xavier et al. (2016b)	643	SCS; DASS-21;	Secondary school pupils	Cross-sectional	15.24; 1.64; 12–18	332/311	*f* – 0.57
							*m* – 0.64
Zeller et al. (2015)	64	SCS; IDAS	Secondary school pupils	Longitudinal	17.5; 1.07; 15–19.	17/47	− 0.23
Stress							
Bluth and Blanton (2014)	67	SCS; PSS	Secondary school pupils	Cross-sectional	16.03; unknown; 15.1–18.7	40/27	−0.70
Bluth and Blanton (2015)	90	SCS; PSS	Secondary school pupils	Cross-sectional	15.1; unknown; 11–18.	50/40	− 0.70
Bluth et al. (2017)	765	SCS-SF; STAI-T; SMFQ; PSS	Secondary school pupils	Cross-sectional	14.6; unknown; 11–19	405/360	− 0.65
Bluth et al. (2016a)	34	CAMM; PANAS; SCS-SF; SMFQ; PSS; STAI	Healthy “stressed” adolescent volunteers	Experimental	14.64; unknown; 14–17	26/8	− 0.49
Bluth et al. (2015)	28	SCS; PSS	Secondary school pupils	Experimental	14.64; unknown; 10–18.	16/12	− 0.73
Bluth, Roberson, Gaylord, Faurot, Grewen, Arzon & Girdler (2016)	28	PSS; SCS; SSAI	Adolescent volunteers	Experimental	14.93; 1.63; 13–18	22/6	− 0.57
Cunha et al. (2013)	651	SCS; DASS	Secondary school pupils	Cross-sectional	15.89; 1.99; 12–19.	321/330	−0.45
Galla (2016)	132	SCS-SF; PSS; CES-DC	Healthy “stressed” adolescent volunteers	Longitudinal	16.76; 1.48; unknown	80/52	− 0.51
Kemper et al. (2015)	29	PSS; CAMS-R	Adolescents with chronic headache	Cross-sectional	14.8; 2.0; unknown	20/9	− 0.71
Tanaka et al. (2011)	117	SCS; CES-D	Adolescents in CPS	Cross-sectional	18.1; unknown; 16–20.	64/53	− 0.33
Marshall et al. (2015)	2448	SCS; GHQ-12	Secondary school pupils	Longitudinal	14.65; 0.45;	1214/1234	− 0.39

Table 2 notes: *BDI* Beck depression inventory (Beck and Steer 1987), *CAMM* child and adolescent mindfulness, measure (Greco et al. 2011), *CAMS-R* cognitive and affective mindfulness scale-revised (Feldman et al. 2007), *CDI* children’s depression inventory (Kovacs 1992), *CES-D* center for epidemiologic studies depression scale (Radloff 1977), *DASS-21* depression, anxiety and stress scale (Lovibond and Lovibond 1995), *GHQ-12* general health questionnaire (Golderberg and Williams 1988), *IDAS* inventory of depression and anxiety symptoms (Watson et al. 2007), *IHSSRLE* the inventory of high-school students’ recent life experiences (Kohn and Milrose 1993), *PANAS* positive and negative affect scales (Watson et al. 1988), *PIY* personality inventory for youth (Lachar and Gruber 1995), *PSS* perceived stress scale (Cohen et al. 1983), *SCS* self-compassion scale (Neff 2003a), *SCS-A* self-compassion scale—adolescent (Cunha et al. 2015), *SCS-SF* self-compassion scale – short form (Raes et al. 2011), *SMFQ* short mood and feelings questionnaire (Angold et al. 1995), *SSAI* Spielberger state anxiety inventory (Spielberger et al. 1970), *STAI-T* Spielberger state-trait anxiety inventory—trait form (Spielberger et al. 1970)

### Measurement of Self-compassion and Psychological Distress

All included studies used the SCS (Neff 2003b), SCS-A (Cunha et al. 2015), or SCS-short-form (SCS-SF, Raes et al. 2011) to measure of self-compassion. The SCS is a self-report measure of beliefs and attitudes based on Neff’s dimensional model of self-compassion (Neff 2003b), composed of 26 items. Recent results from confirmatory factor analysis research affirm that the SCS is a valid and reliable measure of self-compassion in 12- to 18-year-olds, and indicate that the dimensional model of self-compassion can optimise understanding of adolescents’ experience of self-compassion (Cunha et al. 2015), although it should be noted that this study was conducted in a Portuguese sample (Cunha et al. 2015; Neff and McGehee 2010). The SCS-SF has been shown to have near-perfect correlation with the full SCS scale in a sample of English-speaking undergraduate students (Raes et al. 2011). In this analysis, the total SCS/SCS-A/SCS-SF score is reported as the measure of self-compassion in all samples. Table 2 details all the measures used to assess psychological distress outcomes (anxiety, depression, stress) in the included studies.

## Risk of Bias Assessment

The risk of bias within the studies included for meta-analysis was appraised using a bespoke quality assessment tool adapted from Williams et al. (2010). This tool allows raters to grade studies on a range of criteria (see Appendices 1 and 2 for the tool and tool guidance notes). For each criterion, the rater grades qualitatively, answering “Yes”, “No”, “Partially”, or “Cannot Tell”. Table 2 depicts the overall quality rating of studies included in the meta-analysis. In addition, as a supplement to the qualitative assessment, the qualitative ratings were ascribed a numerical value: “Yes” = 2, “Partially” = 1; “No” = 0. Cannot Tell” = 0. Where a criterion was not applicable (“N/A”), no numerical value was applied. These numerical ratings were then added to create a total. A calculation to determine the degree to which a study met its full potential value was then undertaken, and is expressed as a percentage of the number of items given a numerical rating. Independent rating of studies’ risk of bias had a Cohen’s kappa of 0.71 prior to consensus discussion, indicating acceptable reliability.

## Analytic Procedure

### Effect Size Coding

Where stated, effect sizes (*r* values) were directly reported. For studies reporting linear regression data, the standardised regression coefficient (*β* value) was extracted and used as an indicator for effect size (Nieminen et al. 2013).

### Independence of Effect Sizes

Eight studies reported effect sizes for the relationship between self-compassion and multiple psychological distress outcomes (Barry et al. 2015; Bluth et al. 2017; Cunha et al. 2013; Galla 2016; Kemper et al. 2015; Neff and McGehee 2010; Tanaka et al. 2011). Two studies reported separate effect sizes for the relationship between self-compassion and anxiety (Xavier et al. 2016b) and depression (Castilho et al. 2017). Multiple reports of effect sizes within the same study violate assumptions of independence in meta-analytic modelling. Therefore, for studies which reported more than one outcome measure of psychological distress, and for the two studies which reported separate outcome effect sizes by gender, the primary meta-analysis was repeated six times substituting each outcome in turn. There were no differences in overall meta-analytic estimates identified by this process.

### Meta-analytic Model

Analyses were conducted in RStudio (RStudio Version 3.2.2) using the ‘metafor’ (Viechtbauer 2010) and ‘meta’ packages (Schwarzer 2007). The a priori prediction was that identified studies would be heterogeneous across multiple variables. As fixed-effects meta-analytic modeling would inflate the Type 1 error rate, random effects analyses were conducted, using the inverse variance method (Deeks et al. 2001), using DerSimonian Laird (DerSimonian and Laird 1986) estimators for between-study variance. Correlations were converted for meta-analytic estimates using Fisher’s *Z* transformations. The *Q* statistic was used to assess heterogeneity of effect sizes. The *I* ^2^ statistic was used to estimate the total variance due to between-study variance (${I}^2=100\%\frac{\mathrm{Q}-\mathrm{df}}{\mathrm{Q}}$, with *Q* as the statistic defining heterogeneity, and df as the degrees of freedom). Higgins et al. (2003) suggested that *I* ^2^ values of 0, 25, 50, and 75% indicate zero, low, moderate, and high heterogeneity, respectively.

### Publication Bias

As non-significant findings are less likely to be published, mean effect sizes may be exaggerated in the literature. To assess for publication bias, we conducted visual analysis of funnel plots of sample size (standard error) against reported effect size (Fisher’s z). Where there is no publication bias, the funnel plot forms a symmetrical shape. Larger samples collect around the mean effect size, with more dispersal being observed in smaller samples.

In addition to visual analysis of funnel plots, trim-and-fill analysis (Duval and Tweedie 2000) was conducted in order to account for the effect of publication bias on the overall effect sizes of this meta-analysis. Trim-and-fill analysis formalises the qualitative assessment of a funnel plot. In this process, smaller studies are temporarily removed (“trimmed”) from the data set in order to create a symmetrical distribution of data, from which the “true” centre (mean) of the plot is estimated. Once the true mean is identified, the trimmed studies are replaced, along with theoretical counterparts which allow the true mean of the plot to remain (the “fill” stage). Trim-and-fill analysis therefore provides an estimate of the number of studies missing due to publication bias.

## Results

The total sample size of the included studies was *N* = 7049, with 47.7% male (*N* = 3365), 50.62% female (*N* = 3565), and no gender data recorded for the remaining 1.7% (*N* = 119). Information regarding participants’ average age (15 years and 6 months; *N* = 7049) was obtained for all included studies. Participants’ age range (10 to 19 years) was made available for 16 of the included studies. Eleven studies originated from the USA, two from Canada, three from Portugal, one from Australia, one from the UK, and one from Israel. Thirteen studies used a cross-sectional design, four longitudinal, and two experimental (from which pre-intervention data were extracted for inclusion in this meta-analysis). See Table 2 for a summary of study characteristics.

### Reported Effect Size for Self-compassion and Psychological Distress Correlations

**Fig. 2:**
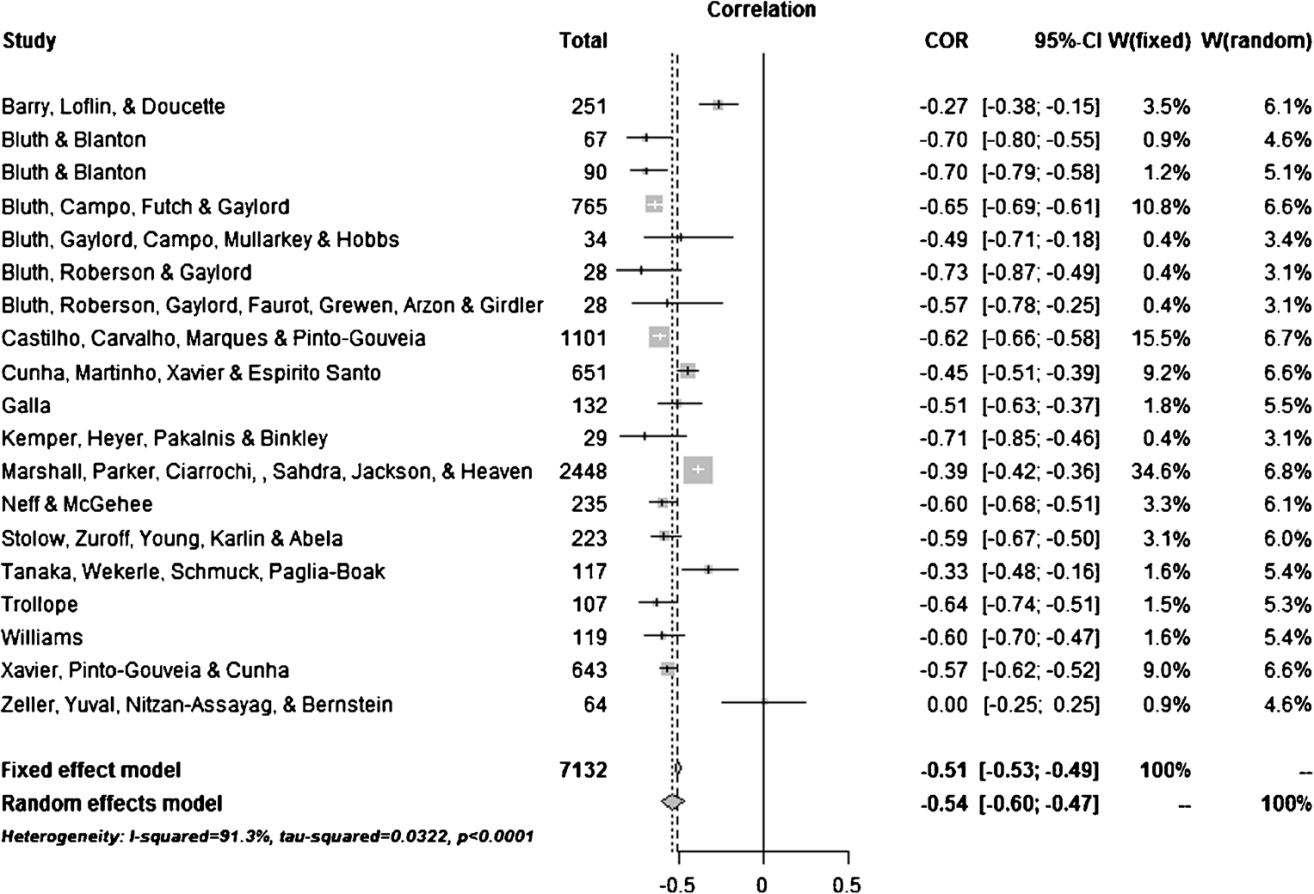
Forest plot of initial meta-analysis of 19 studies

Table 3 displays the summary statistics for the meta-analytic models. The combined uncorrected random effects estimate for the relationship between self-compassion and psychological distress was *r* = − 0.55 (95% CI = − 0.61 to − 0.47, Z = −1 2.78; *p* = < 0.0001; Fig. 2). This corresponds to a large effect size (Cohen 1992), indicating that higher levels of self-compassion were significantly related to lower levels of psychological distress. Observation of the forest plot (see Fig. 2) showed that the majority of included studies reported a moderate to large effect size for the correlation. For the overall sample the effects were significantly heterogeneous (*Q* = 213.99, *p* = < 0.0001), with an *I* ^2^ value of 91.6, indicating that 92% of effect size variance could be attributed to study variance.

**Table 3:** Meta-analyses of relationship between self-compassion and psychological distress (random effects models)

Random effects model	*n*	*N*	Mean effect size *r*	95% CI	*Z*	*p* value	*I* ^*2*^
All studies	19	7132	− 0.54	− 0.60; − 0.47	− 12.91	< 0.0001	91.3
Sensitivity analyses							
All studies including anxiety effects	7	1993	− 0.49	− 0.61; − 0.34	− 5.71	< 0.0001	91.7
All studies including depression effects	13	4437	− 0.52	− 0.57; − 0.46	− 14.16	< 0.0001	83.0
All studies including stress effects	11	4389	− 0.56	− 0.65; − 0.47	− 9.49	< 0.0001	90.8

Table 3 notes: *n* number of studies, *N* total sample size, *mean effect size r* average uncorrected correlation, *95% CI* lower and upper limits of 95% confidence interval for uncorrected correlations, *P value* statistical significance, *I* ^2^ study variance

### Sensitivity Analysis

Seven studies included in the meta-analysis reported multiple measures of psychological distress (anxiety, depression, and stress). Sensitivity analyses were conducted to assess whether the use of different measures of psychological distress had a significant impact on the overall effect size for the entire dataset. Each study contributed one effect size to the analysis. The data reported in Table 3 indicates that the mean effect sizes and 95% confidence intervals for these analyses provided similar results to the main analysis, suggesting that results were not affected by which psychological distress measure was used.

### Publication Bias

An asymmetric distribution of study findings was identified, indicating that publication bias or systematic differences between smaller and larger studies is likely to be present. However, it should be noted that a symmetrical distribution with such a small sample size would be unlikely (Sterne and Egger 2001). The forest plot in Fig. 2 identifies these outliers as Barry et al. (2015) and Zeller et al. (2015).

A linear regression test of funnel plot asymmetry (Egger’s test) was conducted on the pre-trim-and-fill meta-analytic data. Egger’s test (*B* = − 1.45, SE = 1.31) indicated that the findings were not significantly influenced by small study effects or other selection biases.

To account for potential “missingness” of data in the meta-analytic sample, trim-and-fill analysis was conducted on the total sample of studies (examining the effect size of total self-compassion score on overall psychological distress). Trim-and-fill analysis indicated that there were no studies missing from the sample the estimated correlation between self-compassion and psychological distress remained at − 0.55; the post-trim-and-fill confidence interval did not contain zero, and therefore the effect size is considered reliable. The overall effect size remains “large” according to Cohen’s convention (Cohen 1992). The post-trim-and-fill data were significantly heterogeneous (*Q* = 105.31, *p* = < 0.0001), with an *I* ^2^ value of 85.8, indicating that 90.6% of effect size variance could be attributed to study variance.

### Outlier Analysis

A further supplementary analysis was undertaken to model the effects of the two outlier studies on the main dataset. Removing the outlier studies (Barry et al. 2015; Zeller et al. 2015) and re-running the model with *n* = 17 studies generated a combined uncorrected random effects estimate for the relationship between self-compassion and psychological distress, which was *r* = − 0.59 (95% CI = − 0.64 to − 0.52, *Z* = − 14.00; *p* = < 0.0001), indicating a large effect size. Consistent with the main analyses, the effects were significantly heterogeneous (*Q* = 172.3, *p* = < 0.0001, *I* ^2^ = 90.7), with 91% of effect size variance attributable to study variance.

### Identification of Sources of Heterogeneity

To assess for sources of heterogeneity in the main meta-analytic model, meta-regression analyses were conducted to assess for the possible impact of age, gender, and study bias variables. Meta-regression was conducted on all 19 studies, to assess for the impact of age on the self-compassion/psychological distress relationship. Findings indicated that age had a significant relationship with the self-compassion/psychological distress effect sizes (*β* = 1.34, 95% CI = 1.16 to 1.52, *p* = 0.0001), whereby the strength of the self-compassion/psychological distress relationship reduced with increased age.

With regard to gender, three studies were excluded from the meta-regression as one study sample was male-only (Barry et al. 2015), and two reported self-compassion and psychological distress outcomes broken down by gender (Castilho et al. 2017; Xavier et al. 2016b). Therefore, meta-regression was conducted on *n* = 16 studies, to assess for the impact of gender on the self-compassion/psychological distress relationship. Findings indicated that gender did not have a significant relationship with the self-compassion/psychological distress effect sizes (*β*= − 0.0067, 95% CI = − 0.017 to 0.0033, *p* = 0.187).

A final meta-regression analysis (*n* = 19) was conducted, to ascertain whether risk of bias within individual studies (see below) accounted for any variance in the self-compassion/psychological distress relationship. Risk of bias was found to be significantly related to the strength of the self-compassion/psychological distress relationships reported in the sampled studies (*β* = 1.37, 95% CI = 1.18 to 1.56, *p* = 0.0001). Findings showed that the lower risk of bias in a study, the larger the effect size for the negative correlation between self-compassion and psychological distress.

### Quality Assessment

Thirteen studies showed low risk of bias in reporting of cohort demographics, and five reported a reasonable degree of information. Due to the inclusion criteria, all studies in the final sample used the SCS (Neff 2003b), SCS-A (Cunha et al. 2015), or SCS-short-form (SCS-SF, Raes et al. 2011), and at least one valid measure of psychological distress (anxiety, depression, stress; see Table 2). There was mixed quality in the domain of control of potential confounding variables, with 11 studies undertaking stringent methods to control identified confounds in data analysis. However, three studies did not report such measures, and five studies gave only partial detail (see Table 4). Four studies were rated in the 80–100% category indicating low risk of bias. Eleven studies were rated in the 60–79% category, indicating moderate risk of bias. Four studies rated at 50% or below, indicating high risk of bias, as regards the rating in reference to this particular review.

**Table 4:** Risk of bias (ratings assessed using the adapted AHRQ tool)

Authors	Design	Unbiased selection?	Min baseline diff?	Sample size calc?	Cohort description?	SC validated measure?	Psy distress validated measure?	Blinded outcome assessment?	Adequate follow-up?	Missing/drop-out data	Analysis controls for confounds?	Appropriate analysis?	Total score	Percentage	Risk descriptor
Barry et al. (2015)	Cross-sectional	No (0)	N/A	N/A	Partially (1)	Yes (2)	Yes (2)	N/A	N/A	Can’t tell/no (0)	No (0)	Yes (2)	7	50%	High
Bluth and Blanton (2014)	Cross-sectional	No (0)	N/A	N/A	Yes (2)	Yes (2)	Yes (2)	N/A	N/A	Can’t tell/no (0)	Partially (1)	Yes (2)	9	64%	Moderate
Bluth and Blanton (2015)	Cross-sectional	Partially (1)	N/A	N/A	Yes (2)	Yes (2)	Yes (2)	N/A	N/A	Can’t tell/no (0)	Yes (2)	Yes (2)	11	79%	Moderate
Bluth et al. (2017)	Cross-sectional	Yes (2)	N/A	N/A	Yes (2)	Yes (2)	Yes (2)	N/A	N/A	Partially (1)	Yes (2)	Yes (2)	13	93%	Low
Bluth et al. (2016a)	Experimental	No (0)	Partially (1)	Yes (2)	Yes (2)	Yes (2)	Yes (2)	No (0)	N/A	Can’t tell (0)	Yes (2)	Yes (2)	13	65%	Moderate
Bluth et al. (2015)	Experimental	Partially (1)	N/A	N/A	Partially (1)	Yes (2)	Yes (2)	N/A	N/A	Can’t tell/no (0)	Partially (1)	Yes (2)	9	64%	Moderate
Bluth, Roberson, Gaylord, Faurot, Grewen, Arzon & Girdler (2016)	Cross-sectional	Partially (1)	N/A	Can’t tell/no (0)	Yes (2)	Yes (2)	Yes (2)	N/A	N/A	Yes (2)	Yes (2)	Yes (2)	13	81%	Low
Castilho et al. (2017)	Cross-sectional	Yes (2)	N/A	No (0)	Partially (1)	Yes (2)	Yes (2)	Can’t tell (0)	N/A	Yes (2)	Partially (1)	Yes (2)	12	67%	Moderate
Cunha et al. (2013)	Cross-sectional	Yes (2)	N/A	N/A	Partially (1)	Partially (1)	Partially (1)	N/A	N/A	Can’t tell/no (0)	Partially (1)	Yes (2)	8	57%	High
Galla (2016)	Longitudinal	No (0)	N/A	Yes (2)	Yes (2)	Yes (2)	Yes (2)	N/A	Yes (2)	Partially (1)	Yes (2)	Yes (2)	15	83%	Low
Kemper et al. (2015)	Cross-sectional	Partially (1)	N/A	No (0)	Yes (2)	Yes (2)	Yes (2)	No (0)	N/A	Can’t tell (0)	No (0)	Yes (2)	9	50%	High
Marshall et al. (2015)	Longitudinal	Partially (1)	N/A	N/A	Partially (1)	Yes (2)	Yes (2)	N/A	Partially (1)	Partially (1)	Yes (2)	Yes (2)	12	75%	Moderate
Neff and McGehee (2010)	Cross-sectional	Partially (1)	Partially(1)	Can’t tell (0)	Yes (2)	Yes (2)	Yes (2)	Can’t tell (0)	N/A	Can’t tell (0)	Yes (2)	Yes (2)	12	60%	Moderate
Stolow et al. (2016)	Longitudinal	Yes (2)	N/A	Can’t tell (0)	Yes (2)	Yes (2)	Yes (2)	Can’t tell (0)	Partially (1)	Partially (1)	Yes (2)	Yes (2)	14	70%	Moderate
Tanaka et al. (2011)	Cross-sectional	No (0)	N/A	N/A	Partially (1)	Yes (2)	Yes (2)	N/A	N/A	Yes (2)	Yes (2)	Yes (2)	11	79%	Moderate
Trollope (2009)	Cross-sectional	Partially (1)	N/A	N/A	Yes (2)	Yes (2)	Yes (2)	N/A	N/A	Yes (2)	Yes (2)	Yes (2)	13	93%	Low
Williams (2013)	Cross-sectional	Partially (1)	N/A	N/A	Partially (1)	Yes (2)	Yes (2)	N/A	N/A	Yes (2)	Partially (1)	Yes (2)	11	79%	Moderate
Xavier et al. 2016a	Cross-sectional	Yes (2)	N/A	No (0)	Partially (1)	Yes (2)	Yes (2)	No (0)	N/A	Can’t tell (0)	Yes (2)	Yes (2)	11	61%	Moderate
Zeller et al. (2015)	Longitudinal	No (0)	N/A	N/A	Yes (2)	Yes (2)	Yes (2)	N/A	Partially (1)	Can’t tell/no (0)	Can’t tell (0)	Yes (2)	9	56%	High

## Discussion

This meta-analysis examined the relationship between self-compassion and psychological distress in adolescents, and found that these factors were inversely correlated with a large effect size; therefore higher levels of self-compassion were associated with lower levels of distress. These findings replicate those reported in adult populations (MacBeth and Gumley 2012), although the data from adolescent samples contains greater degree of variance. Nine of the 19 included studies reported effect sizes for the relationship between self-compassion and multiple psychological distress outcomes. Sensitivity analyses found that substituting these individual effect sizes did not significantly alter the mean estimate and confidence intervals for the overall effect size. Studies using multiple measures of psychological distress violated the assumption of independence; therefore, further analysis of self-compassion related to specific distress outcomes was deemed inappropriate, and remains an area for future investigation.

Findings from meta-regression analysis indicated that age had a significant relationship to the self-compassion/psychological distress correlation (*N* = 7049) with the magnitude of effect weakening as a function of age—with older adolescents reporting a weaker association between higher levels of self-compassion lower levels of distress compared with younger adolescents. It seems that age or stage of adolescence may be particularly important to examine when considering the development of self-compassion (it must be noted that defining the parameters of adolescence is a challenge in research, and that division of the adolescent period into age-related stages is a somewhat arbitrary exercise).

According to Gilbert (2009)—who frames compassion as a product of human social evolution, with roots in the capacity to engage in mentalisation and form rewarding relationships with others—adolescence is a time of significant biopsychosocial change, and as such adolescents’ sympathetic nervous systems are highly ‘primed’ for activation, thus elevating risk for development of psychopathology (Gilbert and Irons 2009). Adolescents have a magnified need to exist positively in others’ regard. This need may be a source of increased self-criticism, self-judgement, and shame (Gilbert and Irons 2009)—processes which have been directly related to symptoms of psychopathology (Reimer 1996). Increased sympathetic nervous system reactivity and increased demand on abstract mental processing and social interaction skills may provide an explanation for the evidence that self-compassion decreases with age—in female adolescents at least (Bluth and Blanton 2015), although more detailed investigation of the way these factors inter-relate is required.

Findings from meta-regression analyses showed that gender did not have a significant relationship to the self-compassion/psychological distress correlation in this meta-analytic sample of 16 studies (*N* = 5054). One possible explanation for the findings from this meta-analysis (that age, but not gender, bears a significant relationship to self-compassion/psychological distress correlations in adolescents) is that these factors are interactive. In adolescent samples, some researchers have identified an interaction effect of gender and age on self-compassion, with older female adolescents having lower levels of self-compassion than younger females or males of any age (Bluth and Blanton 2015; Bluth et al. 2016a; Muris et al. 2016). It may be that the development of certain cognitive abilities typical of the mid-to-late adolescent period (such as the “imaginary audience”; Elkind 1967) paired with greater cultural judgement of females (certainly within Western societies; Grant 2013), results in adolescent females’ increased vulnerability to the development anxiety, depression, and stress (Grant 2013), and the internalisation of a less compassionate manner of relating to themselves (Neff and Vonk 2009). However, findings in adult populations indicate that this putative effect of gender lessens over time (Yarnell et al. 2015), highlighting a need for further investigating regarding the interaction of age and gender in the experience of self-compassion.

Analyses also indicated that as risk of bias fell within the sampled studies, the inverse relationship between self-compassion and psychological distress became more marked (19 studies, *N* = 7049). This finding is encouraging, as it provides additional evidence that the overall results of this meta-analysis are robust, and highlight the value to the field of conducting methodologically rigorous studies of self-compassion. Unfortunately, another potential source of heterogeneity—socio-economic status (SES)—was not consistently reported, thus limiting us from including it in the moderator analyses. Some findings have indicated that poverty and ethnic minority status in developed countries are positively related to level of self-compassion, and that self-compassion is a moderating mediator between low income and academic success (Conway 2007).

Whilst the research base examining the impact of SES on the experience of self-compassion is limited, early indications suggest that it is a pertinent factor which may explain some of the heterogeneity in the results of this meta-analysis (Stellar et al. 2012; Yarnell et al. 2015). Investigation of the role of developmentally appropriate SES variables (e.g., educational level and family income) could be a useful adjunct for future research. Likewise, if there is an aspiration to increase the breadth of self-compassion as a tool for building resilience (Kieling et al. 2011), it is necessary that research be conducted using samples from both high and low/middle income countries.

### Self-compassion Interventions for Adolescents

Adolescence is a critical period characterised by vulnerability to psychological distress, and is therefore an important time for promotion of psychological well-being and early mental health intervention, in order to safeguard against the development of mental health issues (Xavier et al. 2015). Effective mental well-being promotion and early intervention in this stage of life can prevent substantial personal distress and social cost (Patel et al. 2007). It is therefore imperative to identify factors which will be most effective in promoting resilience and well-being in this population. Muris and Meesters (2014) have highlighted the potential utility of self-compassion interventions as a method of buffering against the formation of negative self-conscious emotional and cognitive styles (which are linked to development of anxiety and depression) in youth.

Bluth and Eisenlohr-Moul (2017) report the outcome of a small-scale study of an 8-week self-compassion group programme for adolescents, with a 6-week follow-up period. Bluth and Eisenlohr-Moul (2017) found that participants’ level of perceived stress reduced to a significant degree post-intervention and at follow-up. Resilience was found to have increased significantly at follow-up, and gratitude and curiosity increased significantly post-intervention and at follow-up. There was a non-significant decrease in anxiety and depression symptoms post-intervention and at follow-up.

Similarly, a small-scale pilot of a 6-week mindful self-compassion programme for non-clinical adolescents (Bluth et al. 2016a) has found that those who completed the programme reported increased levels of self-compassion and life satisfaction, as well as significantly lower levels of depression than adolescents in the control group. Mindfulness and self-compassion were both found to predict lower levels of anxiety, depression, perceived stress, and low mood in adolescents in the intervention group (Bluth et al. 2016a).

Overall, the findings of this meta-analysis support the hypothesis (and early research findings) that, as in adult populations, self-compassion is a potentially important construct in understanding and treating adolescent mental health issues.

### Measuring Self-compassion and Mindfulness

The data provided within the studies included in this meta-analysis did not support an investigation of the potential differential effects of mindfulness and self-compassion on psychological distress outcomes. Existing research has reported that self-compassion and mindfulness may have different (Bluth et al. 2016a; Galla 2016) and complementary (Bluth and Blanton 2014; Edwards et al. 2014) roles in reducing psychological distress symptoms and elevating well-being in youth; findings which echo those in adult samples (Baer et al. 2012; Birnie et al. 2010; Hollis-Walker and Colosimo 2011; Van Dam et al. 2011).

If we are to understand these relationships more accurately in all age groups, researchers may need to measure these constructs independently. Independent measurement of mindfulness and self-compassion requires careful definition of each construct, particularly with regard to whether mindfulness is to be considered a subcomponent of self-compassion, or a more “global” construct independent of the mindfulness in self-compassion. If researchers are content to define mindfulness as a subcomponent of self-compassion, as in Neff’s dimensional model, it seems reasonable to suggest that the current drive towards reporting SCS subscale outcomes in research (Neff 2016) is an appropriate way of better understanding the differential roles of mindfulness, self-kindness, and common humanity (and their “negative” dimensional counterparts) in both adolescent and adult samples.

However, Neff’s model explicitly recognizes mindfulness both as a constituent part of compassion, and also as an independent construct with the facility to mediate pathways to emotional well-being (Bluth and Blanton 2014). Neff and Dahm (2013) explain that the mindfulness component of self-compassion is “…narrower in scope than mindfulness more generally” (Neff and Dahm 2013, p20), being focused solely on reviewing negative thoughts and feelings, whereas the broader concept of mindfulness is characterised by awareness of all aspects of experience. With this explicit separation between “global” mindfulness and the mindfulness element of self-compassion already defined in the self-compassion literature, we are led to conclude that future researchers must use independent measures to investigate the differential influence of self-compassion and mindfulness on psychological experience, and the relationship between these two factors. It should be noted that the recommendation to measure self-compassion and “global” mindfulness separately is inextricably linked with the documented difficulties with conceptualizing and measuring mindfulness in a valid and reliable manner in Western science (Grossman 2011). Without robust methods of defining and measuring global mindfulness, it will be challenging to discern if there is any true difference in how self-compassion and global mindfulness relate to psychological well-being and distress outcomes in any population.

### Limitations

With regard to limitations, due to the prevalence of cross-sectional study design, this review was only able to report on the strength of correlation between self-compassion and psychological distress, rather than examining causality in this relationship. Further longitudinal and experimental research must be conducted to explore the direction of relationships between self-compassion and psychological distress outcomes—although some researchers have demonstrated that low self-compassion predicts depression in later life: Krieger et al. (2016) report that in adults with depression, low self-compassion predicts elevated symptoms of depression 6 months later. Raes (2011) reported that, in a non-clinical sample of adults, higher self-compassion predicted greater reduction in depressive symptoms, or smaller increases in depressive symptoms, at 5-month follow-up. Without clarity regarding the nature of these relationships, we cannot be certain that self-compassion is an appropriate factor to harness in psychological interventions.

A further methodological limitation was identified with regard to the risk of bias within the studies included in the meta-analysis. Based on the risk of bias assessment parameters of this review, risk in the majority of studies was moderate (58%; 11 studies) to high (21% four studies), with 21% (four studies) study rated as low risk. To increase the robustness and generalisability of research findings, greater care must be taken in the literature to report participant sampling, cohort description, and methodological design—thus, reducing risk of bias and therefore increasing the reliability and validity of results. Finally, whilst this meta-analysis was able to identify factors which are related to the documented self-compassion/psychological distress inverse correlation (e.g., age and risk of bias within studies), future research examining potential moderating/mediating roles of individual factors (e.g., age) may be merited. This in turn may have implications for psychological well-being promotion and interventions for psychological distress. Research which develops our understanding cross-cultural influences on the development and maintenance of self-compassion, and the role of societal/systemic level factors may advance understanding of how best to foster an environment which nurtures self-compassion, rather than making it the responsibility of the developing individual.

## Appendix 1: Quality Rating for Adolescent Self-Compassion Meta-Analysis

To be used in conjunction with adapted AHRQ checklist notes.

**Study Name:**

**Reviewer:**

**Date:**

**Checked by Lead Researcher:**

**Table:**

**Item**	**Descriptor**	**Decision** (Yes/No/Partially/Can’t Tell)	**Notes**
**1**	**Unbiased Selection of Participant Sample?**		
**2**	***Selection minimizes baseline differences? (controlled studies)***		
**3**	***Sample Size Calculated? (controlled studies and population studies only)***		
**4**	**Adequate description of the cohort?**		
**5**	**Validated method for ascertaining level of self-compassion?**		
**6**	**Validated method for ascertaining psychological distress outcomes?**		
**7**	**Blinded outcome assessment?**		
**8**	***Adequate follow-up period (longitudinal studies only)?***		
**9**	**Missing data/drop-out**		
**10**	***Analysis controls for confounding (in controlled studies and where studies test for predictors/correlates of level of self-compassion)?***		
**11**	**Analytic methods appropriate** ***?***		

## Appendix 2: Quality Rating of Adolescent Self-Compassion and Psychological Distress Outcome Studies

**Adapted from:** Williams et al. (2010). Preventing Alzheimer’s Disease and Cognitive Decline. Evidence Report/Technology Assessment No. 193. (Prepared by the Duke evidence-based practice center under contract No. HHSA 290–2007-10,066-I). Agency for Healthcare Research and Quality: Rockville, MD.

**General instructions**: Grade each criterion as **“Yes,” “No,” “Partially,**” or **“Can’t tell.”**

Where item is not applicable write: **N/A.**

Factors to consider when making an assessment are listed under each criterion. Note that some criteria will only apply to specify types of study.

**Note:** Where a criterion only applies to a specific design, it is in italics.

Definitions:

Self-compassion = level of self-compassion as ascertained by a valid and reliable measure of the construct.

Psychological distress = measures of mood, anxiety and stress as ascertained by valid and reliable measures.

1. **Was the selection of the participant sample unbiased?**

Factors that help reduce selection bias:

- Inclusion/exclusion criteria is clearly described
- Recruitment strategy is clearly described
- The nature of the population (typical or clinical) is clearly detailed

Also: the sample is representative of the population of interest: adolescents.

2. **Selection minimizes baseline differences between samples (controlled studies only)?**

Factors to consider:

- Was selection of the comparison group appropriate? Consider whether comparable participant samples are likely to differ on factors related to the outcome. Matching on key demographics (e.g., gender and population sample type) would be required to minimize bias.
- Did the study investigators do other things to ensure that comparable groups were similar, e.g., by using stratification or matching techniques?

3. **Sample size calculated (for controlled studies and where studies test for predictors/correlates of self-compassion)?**

Factors to consider:

- Did the authors report conducting a power analysis or describe some other basis for determining the adequacy of study group sizes for the primary outcome(s) of interest to us?
- Did the eventual sample size deviate by ≤ 10% of the sample size suggested by the power calculation?

4. **Adequate description of the cohort?**

- Consider whether the cohort is well-characterized in terms of baseline demographics.
- Consider key demographic information such as age, gender and country of origin.
- Inclusion of information regarding education or socio-economic characteristics is also important.

5. **Validated method for ascertaining level of self-compassion?**

Factors to consider:

- Was the method used to ascertain level of self-compassion clearly described? (Details should be sufficient to permit replication in new studies)
- Was a valid and reliable measure used to ascertain level of self-compassion?

6. **Validated method for ascertaining psychological distress outcomes?**

Factors to consider:

- Were psychological distress outcomes assessed using valid and reliable measures? Note that measures that consist of single items of scales taken from larger measures are likely to lack content validity and reliability.
- Were these measures implemented consistently across all study participants?

7. **Blinded outcome assessment?**

- In studies using experimental designs or comparing cohort outcomes, were investigators blind to sample group when assessing outcome data?

8. **Adequate follow-up period (longitudinal studies only)?**

Factors to consider:

- A justification of the follow-up period length is preferable.
- Follow-up period should be the same for all groups
- If differences in follow-up time were present, was this difference adjusted for using statistical techniques?

9. **Missing data/drop-out**

Factors to consider:

- Did missing data from any group exceed 20%?
- In longitudinal studies consider attrition over time as a form of missing data. Note that the criteria of <20% missing data may be unrealistic over longer follow-up periods.
- If missing data is present and substantial, were steps taken to minimize bias (e.g., sensitivity analysis or imputation)?

10. **Analysis controls for confounding (in controlled studies and where studies test for predictors/correlates of level of self-compassion)?**

Factors to consider for controlled studies:

Does the study identify and control for important confounding variables and effect modifiers? These may include demographic and clinical variables.

11. **Were analytic methods appropriate?**

Factors to consider:

- Was the kind of analysis done appropriate for the kind of outcome data (categorical, continuous, etc.)?
- Was the number of variables used in the analysis appropriate for the sample size? (The statistical techniques used must be appropriate to the data and take into account issues such as controlling for small sample size, clustering, rare outcomes, multiple comparison, and number of covariates for a given sample size)
